# Splice site identification using probabilistic parameters and SVM classification

**DOI:** 10.1186/1471-2105-7-S5-S15

**Published:** 2006-12-18

**Authors:** AKMA Baten, BCH Chang, SK Halgamuge, Jason Li

**Affiliations:** 1 Dynamic Systems and Control Research Group, DoMME, The University of Melbourne, Victoria 3010, Australia

## Abstract

**Background:**

Recent advances and automation in DNA sequencing technology has created a vast amount of DNA sequence data. This increasing growth of sequence data demands better and efficient analysis methods. Identifying genes in this newly accumulated data is an important issue in bioinformatics, and it requires the prediction of the complete gene structure. Accurate identification of splice sites in DNA sequences plays one of the central roles of gene structural prediction in eukaryotes. Effective detection of splice sites requires the knowledge of characteristics, dependencies, and relationship of nucleotides in the splice site surrounding region. A higher-order Markov model is generally regarded as a useful technique for modeling higher-order dependencies. However, their implementation requires estimating a large number of parameters, which is computationally expensive.

**Results:**

The proposed method for splice site detection consists of two stages: a first order Markov model (MM1) is used in the first stage and a support vector machine (SVM) with polynomial kernel is used in the second stage. The MM1 serves as a pre-processing step for the SVM and takes DNA sequences as its input. It models the compositional features and dependencies of nucleotides in terms of probabilistic parameters around splice site regions. The probabilistic parameters are then fed into the SVM, which combines them nonlinearly to predict splice sites. When the proposed MM1-SVM model is compared with other existing standard splice site detection methods, it shows a superior performance in all the cases.

**Conclusion:**

We proposed an effective pre-processing scheme for the SVM and applied it for the identification of splice sites. This is a simple yet effective splice site detection method, which shows a better classification accuracy and computational speed than some other more complex methods.

## Background

Advances in the genome sequencing technology have created a vast amount of sequence data and completed genomic sequences. Identification of all genes is one of the major objectives of a genome sequencing project. In eukaryotic genomes, the detection of a coding region also depends on the precise identification of the exon-intron structures. However, the vast length and structural complexity of sequence data makes it a very challenging task. Recent genome analysis shows that the human genome contains approximately 3 billion base pairs and 20,000–25,000 protein-coding genes [1]. However, it was statistically estimated that the number of genes in human genome should be around 100,000 [2]. This difference shows that either a large number of genes are yet to be identified or there are many alternative splicing events yet to be detected [3,4]. Various computational methods have been proposed in the last decade for the identification of eukaryotic genes. Most of those methods perform well to a certain extent and have their own limitations. So despite of many years of intensive research in this area, the overall performance of the gene prediction algorithms is still not satisfactory [5,6].

**Figure 1:**
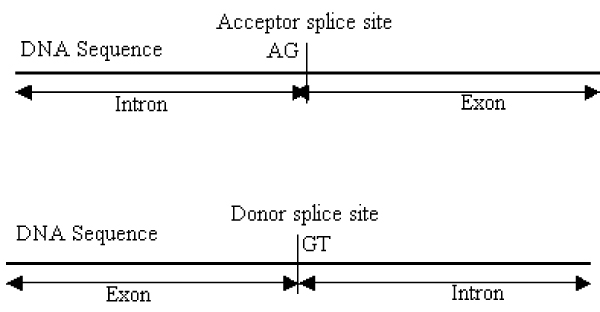
Illustration of acceptor and donor splice sites. Introns usually end with dinucleotides AG and the border between intron and exon in a DNA sequence is termed as acceptor splice site. Introns usually start with dinucleotides GT and the border between exon and intron in a DNA sequence is termed as donor splice site.

Most of the eukaryotic protein coding genes consist of introns and exons. The exons are the protein coding region of a gene and they are interspersed with intervening sequences of introns. Introns are termed as protein non coding regions as their biological significance is not well known yet. The borders between introns and exons are termed as splice sites. The splice site in the upstream part of an intron is called the donor splice site (in the direction 5' to 3') and the downstream part is termed as the acceptor splice site (in the direction 3' to 5'). The acceptor and donor splice sites with consensus AG (correspond to the end of an intron) and GT (correspond to the beginning of an intron) respectively are known as canonical splice sites as shown in Figure 1. These canonical acceptor and donor splice sites are recognized by the major class, or U2-type spliceosome, which is universal to eukaryotes [7]. The non canonical splice sites are those with minor consensus such as GC and AC and are recognized by the minor class or U12-type spliceosome, which may not be present in some organisms [8]. Approximately 99% of the splice sites are canonical AG/GT splice sites [7]. As AG and GT represent possible acceptor and donor splice sites, every AG and GT of a DNA sequence is a candidate acceptor or donor splice site and they need to be classified as either a real (true) splice site or a pseudo (false) splice site.

In eukaryotes, introns are removed from gene transcripts (exons) in a biological process known as pre-mRNA splicing. This is an enzymatic reaction that involves a large multi component ribonucleoprotein catalytic complex known as spliceosome. Mass spectrometry analysis is a technique to identify the spliceosome and splicing factors that participate in the pre-mRNA splicing. In 1999, around 100 splicing factors were identified [8]. However, recent improved and more sensitive mass spectrometers and sample preparation techniques found more than 300 polypeptide that participate in the splicing which may include some more complex mechanism beyond our current knowledge of pre-mRNA splicing mechanism [9,10].

Various computational methods have been developed for splice site detection, and they can be grouped into several categories including the probabilistic approaches [3,6,11-17], the neural network and support vector machine approaches [18-26], the methods based on discriminant analysis [27,28] and the information theoretic approaches [29-31]. These methods are based on seeking the consensus patterns or features and try to identify the underlying relationships among nucleotides in a splice site and the surrounding region. Neural networks and support vector machines (SVM) learn the complex features of neighbourhoods surrounding the consensus di-nucleotides AG/GT by a complex non-linear transformation. Probabilistic models estimate position specific probabilities of splice sites by computing likelihoods of candidate signal sequences. The discriminant analysis uses several statistical measures to evaluate the presence of specific nucleotides, recognizing the splice sites without explicitly determining the probability distributions.

In the past, researchers also attempted to identify splice sites using the weight matrix method (WMM) inspired by the presence of apparent consensus AG and GT in the splicing junctions [19,31]. WMM was also adopted in methods NetPlantGene [20] and NNSplice [18]. Later, Salzberg *et al*. and Zhang *et al*. [13,14] achieved a good splice site prediction accuracy using a linear first order Markov model (MM1, WAM). However, MM1 alone cannot model the complex higher-order dependencies that exist among the nucleotides in both acceptor and donor splice sites. The unavailability of high quality training data encourages researchers to design models which can learn the complex nucleotide dependencies efficiently from the limited available training data. To overcome the limitations of MM1 without a high quality and large training dataset, Burge *et al*. proposed the Genscan [6] method. Genscan is a computational method based on the maximal dependence decomposition (MDD) algorithm, which is a decision tree process that captures dependencies among nucleotides. The MDD is designed to capture the most significant dependencies between adjacent and non-adjacent nucleotides. Even though the Bayes network model [16] and MDD are complex splice site detection methods, they do not produce a dramatic improvement in splice site detection with respect to simpler models that assume dependencies only between adjacent positions. It has been suggested that a significant improvement in the detection of splice sites is possible if one of the base statistical models, such as WMM, MM1, MDD etc., is combined with other signal/content methods [11]. GeneSplicer is a method of this category [11], where second order Markov models (MM2) are combined with MDD. Similarly, Rajapakse and Ho *et al*.[32] introduced a more complex splice site prediction system which combines mostly MM2 and backpropagation neural networks (BPNN). This approach shows better prediction accuracy than Genesplicer but requires longer sequence windows for the training. However, the use of backpropagation neural network is already computationally expensive and with the inclusion of second order Markov models for data pre-processing, the computational complexity increases even more.

WMM, MM1, MM2, and MDD are the most popular methods employed for splice site detection and they require the manual selection of information source. In contrast, machine learning technique such as SVM has the advantage of inferring an optimal classifier from the training data. SVM has been used to classify splice site data as reported in [22-26] with limited success. Mostly, these approaches employ SVM to compute a classification boundary between true and false (pseudo) splice sites. For this, a candidate splice site sequence is represented as a feature vector, with each feature containing some information about the candidate splice site and its context in the sequence.

Markov models are being used for extracting sequential relationships that enables the inclusion of biological knowledge to differentiate compositional differences of nucleotides in a splice site and it's surrounding regions [11-13,32,33]. In this work, it is shown that a simple MM1 can be effectively combined with a classifier such as SVM, to extract sequential information with a reduced computational complexity. Several simulations have been performed with well-known and publicly available splice site data sets. Results show that our proposed MM1-SVM method produces a better classification performance and identifies acceptor and donor splice sites more efficiently than other existing methods.

## Results

### Best pre-processing model selection

We used several pre-processing methods including MM0, MM1, WMM0, and WMM1 with SVM classifiers for the identification of splice site. The goal is to identify the best pre-processing method that enhances the classification accuracy of the SVM. We used NN269 acceptor and donor dataset to compare the prediction accuracies of MM0-SVM, MM1-SVM, WMM0-SVM, and WMM1-SVM methods. As MM0 and WMM0 imply the same model, we refer the integration of these two models with SVM as WMM0/MM0-SVM.

**Figure 2:**
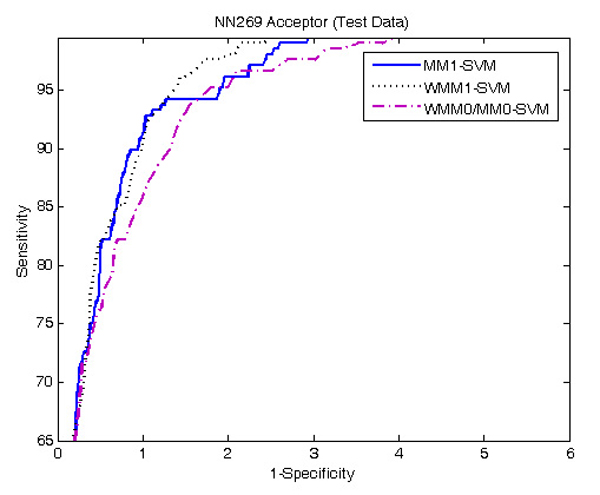
ROC curve showing the comparison of performance between methods MM1-SVM, WMM0/MM0-SVM, and WMM1-SVM using NN269 acceptor dataset. MM1-SVM and WMM1-SVM performs almost equally well. WMM0/MM0-SVM performs worst among the three methods.

**Figure 3:**
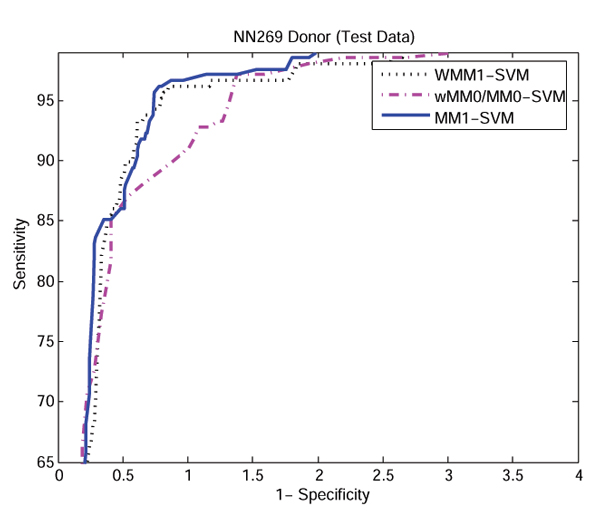
ROC curve showing the comparison of performance between methods MM1-SVM, WMM0/MM0-SVM, and WMM1-SVM using NN269 donor dataset. MM1-SVM and WMM1-SVM performs almost equally well. WMM0/MM0-SVM performs worst among the three methods.

Figure 2 and 3 show the ROC (receiver operating curve) analysis of the models WMM0/MM0-SVM, MM1-SVM, and WMM1-SVM using NN269 acceptor and donor dataset. We observed that MM1-SVM and WMM1-SVM are the best predictive models in the identification of both acceptor and donor splice sites, and the performance of WMM0/MM0-SVM is the worst. In this study, the MM1-SVM model is used as our main method for splice site detection.

### Classification performance comparison

**Figure 4:**
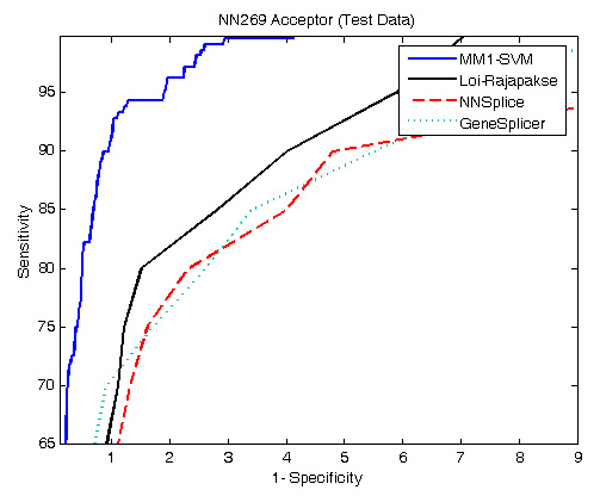
ROC curve showing the comparison of performance between MM1-SVM, Loi-Rajapakse method, NNSplice, and GeneSplicer using NN269 acceptor dataset. MM1-SVM produces the best performance while Loi-Rajapakse method produces the second best performance. NNSplice and GeneSplicer produce the worst performance in this case.

**Figure 5:**
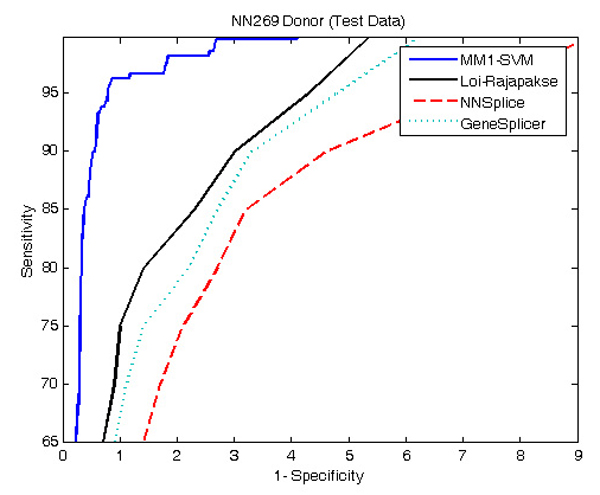
ROC curve showing the comparison of performance between MM1-SVM, Loi-Rajapakse method, NNSplice, and GeneSplicer using NN269 donor dataset. MM1-SVM produces the best prediction accuracy. Loi-Rajapakse method produces the second best performance while NNSplice produces the worst performance.

Figure 4 and 5 show the comparison of performance between the proposed MM1-SVM, Loi-Rajapakse [32] method, GeneSplicer [11], and NNSplice [18] using NN269 dataset. The standard sensitivity and specificity measures are employed for the comparison purpose. As shown in both Figures 4 and 5, MM1-SVM is clearly the superior model for the identification of both acceptor and donor splice sites. In acceptor splice site prediction, Loi-Rajapakse method produced the second best performance. Our proposed method MM1-SVM outperforms all the methods for the identification of donor splice sites. NNSplice produces the worst performance in this case. The maximum sensitivity and specificity values for MM1-SVM are 96% and 97% for the acceptor splice site prediction and 97% and 98% for the donor splice site prediction.

**Figure 6:**
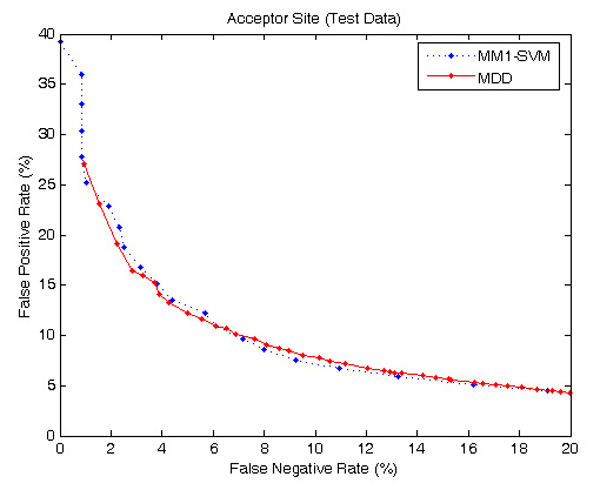
ROC curve showing the comparison of performance between MM1-SVM and MDD using DGSplicer acceptor dataset. MDD performs almost equally as good as MM1-SVM.

**Figure 7:**
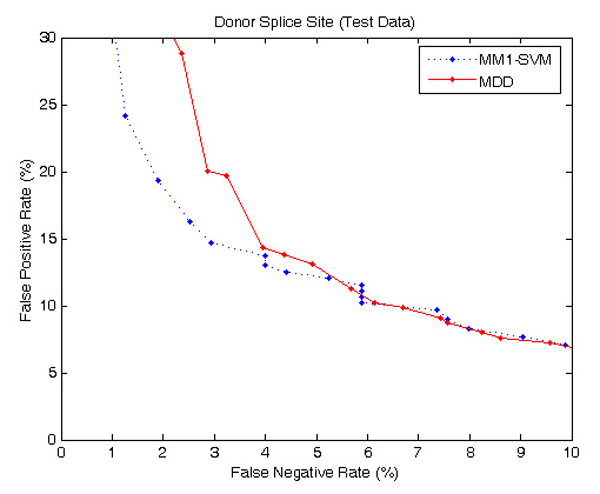
ROC curve showing the comparison of performance between MM1-SVM and MDD using DGSplicer donor dataset. MM1-SVM performs better than MDD.

To further verify the prediction accuracies of the MM1-SVM method we used a larger DGSplicer dataset, and compared the performance with MDD method [6] as shown in Figures 6 and 7. Both MDD as well as MM1-SVM perform well for acceptor splice sites; however, in the identification of donor splice sites, MM1-SVM shows a superior performance.

## Discussion

In this study, we presented a new splice site detection method that can identify acceptor and donor splice sites in DNA sequences. Our proposed MM1-SVM method shows a better prediction accuracy in all cases when tested with two large and well curated dataset.

Markov models, WMMs, and classifiers such as SVMs are well studied methods and have been successfully applied not only in the areas of splice site detection but also in other areas in bioinformatics. We observed that the performance of these methods as standalone applications is not satisfactory. However, their performance may be improved when they are integrated with each other. Even though SVM is a well established algorithm and it is popular in classification and regression tasks, its performance in genome signal identifications (e.g. splice site) is not as good as expected. This is largely due to the way genome sequence data is presented to them. Mostly, sequence data are presented directly to a classifier using a binary encoding technique [34]. It was observed in our study that classifiers cannot properly discriminate true and false signals based on the plain sequence data. This suggets that classifiers such as SVM require more information than plain sequence data to make a satisfactory classification. We showed that a probabilistic encoding scheme of genome sequence data can help SVM to achieve better performance due to the added nucleotide dependency information. Three different probabilistic pre-processing schemes are presented in this paper namely, MM1, WMM0/MM0, and WMM1. All the pre-processing models help to improve the performance of the SVM due to the added nucleotide dependency and positional information. Among all the pre-processing models, MM1 is observed as most useful for SVM. A MM1 models the first order sequential relatioships of nucleotides in terms of probabilistic parameters and a SVM takes these parameters as its input. Through its highly complex non-linear transformation, a SVM transforms the lower order sequential relationships into a higher order one and produces the prediction. WMM1 preprocessing also improves the performance of a SVM. However, the performance of WMM1-SVM is not as good as MM1-SVM because WMM1 only takes into account the observed frequencies of pair of nucleotides and do not necessarily model the dependencies between nucleotides. Even though it has been suggested that a method which is able to capture higher order sequential relationships would perform better, its successful implementation is largely dependent on the availibility of large dataset as as they require the estimation of a large number of parameters. Moreover, the modeling of higher order dependency is also computationally expensive as the computational cost increases exponentially with the increase of the order of a Markov model. In this paper we showed that the integration of low order Markov models such as a MM1 with the classifiers such as a SVM can in effect produce a higher order Markov model. However, the tuning of SVM parameters is still a challenge if the size of training dataset is not balanced between true and false data and there is a chance that a SVM would overfit the data. We have ensured that the SVM is not overfitted in this study by using the cross validation technique. In this study, we mainly used SVM with a polynomial kernel. However, SVM with linear and RBF kernels are also implemented for performance comparison. This comparison can be found in the Additional file: 1.

Our proposed method is faster than the Loi-Rajapakse [32] method as it requires calculation of fewer Markovian parameters (refer to the method section). Also, from our simulations (not reported in this paper) with Radial basis functions network (RBFN), standalone SVM (without MM1 pre-processing), and standalone backpropagation network (BPNN), we concluded that the proposed method is the fastest.

In this paper we only studied the identification of canonical splice sites which forms around 99% of all splice sites in eukaryotes. However this method can also be adjusted to identify the remaining and less frequent 1% non-canonical splice sites as well. The accuracy of splice site prediction of our proposed method suggests that this method can be useful in identifying genes in genomic sequences. The proposed method can be applied to genome sequence data for the identification of regulatory elements such as gene translation initiation sites [35]. However, the size of the data may need the use of training data reduction algorithms [36] unless large scale computing resources are used. If the SVM involves a RBF kernel, it is also possible to interpret the trained classifier as a rule based system [37].

## Methods

### Overview of the proposed method

**Figure 8:**
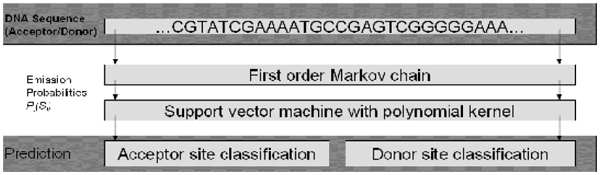
Overview of the model. The input DNA sequence data is pre-processed by a first order Markov model which generates probabilistic parameters. A SVM with polynomial kernel takes these parameters as its input for the splice site classification.

The proposed method consists of two stages: a first order Markov model (MM1) pre-processing and a support vector machine (SVM) with polynomial kernel. In this study, a MM1 aims to learn the conserved sequence pattern at upstream and downstream regions surrounding the splice site motifs (GT-AG). Firstly the MM1 processes the input sequence data and generates some position specific probabilistic parameters (emission probabilities). These probabilistic parameters are then fed into a SVM with polynomial kernel, whose outputs are used to make prediction as illustrated in Figure 8.

### Markov model pre processing of splice site data

#### First order Markov model

Each nucleotide in a DNA sequence corresponds to a state in the Markov chain used, whose observed state variables are drawn from the alphabet Ω_*DNA *_= {*A*, *C*, *G*, *T*}. Let us define an arbitrary sequence of length *l *:{*s*_1_, *s*_2_,...., *s*_*l*_}, where {*s*_*i *_∈ {*A*, *C*, *G*, *T*}, ∀*i*∈{1,....,*l*}, then the nucleotide *s*_*i *_is a realization of the *i *th state variable of a Markov chain, and state transition is only allowed from state *i *to its adjacent state *i *+ 1. Hence, the model consists of states ordered in a series. It evolves from state *s*_*i *_to *s*_*i*+1 _and emits symbols from the alphabet Ω_*DNA*_, where each state is characterized by a position-specific probabilistic parameter. Assuming a Markov chain of order *k*, the likelihood of a sequence given the model is:



where the Markovian probability *P*_*i*_(*s*_*i*_) = *P*(*s*_*i*_|*s*_*i*-1_, *s*_*i*-2_,....,*s*_*i*-*k*_) denotes the conditional probability of a nucleotide at location *i *given the *k *predecessors. Such a model is characterized by a set of parameters:

{*P*(*s*_*i*_|*s*_*i*-1_,....,*s*_*i*-*k*_): *s*_*i*_,*s*_*i*-1_,....,*s*_*i*-*k *_∈ Ω_*DNA*_,*i *= 1,2,...,*l*}.

In the proposed method, a MM1 is used to model a set of nucleotides in a sequence. The Markovian parameters are expressed interms of position-specific first order conditional probabilities (*k *= 1):

*P*_*i*_(*s*_*i*_)=*P*(*s*_*i*_|*s*_*i*-1_).     (2)

The model is then characterized by the set of parameters: {*P*(*s*_*i*_|*s*_*i*-1_):*s*_*i*_, *s*_*i*-1 _∈ Ω_*DNA*_,*i *= 1,2,...*l*}.

#### Higher order Markov model

It is generally accepted that higher order Markov models are more efficient in capturing possible interactions among nucleotides surrounding the splice sites [6,38]. However, a larger set of training sequences is required for higher order Markov models. For a *k th*-order Markov model, the training set must provide coverage of all possible subsequences of nucleotides of length *k *+1 at every sequence position for the estimation of 4^*k*+1 ^Markovian parameters. The required number of training samples increases exponentially with the order of a Markov model. With the limited amount of training data available and the high computational complexity, it often makes the implementation of such models practically impossible.

Loi-Rajapakse [32] suggested that the sequence should be divided into upstream, signal, and downstream segments. The signal segment is modelled by a MM1, whereas, the downstream and upstream segments are modelled by two MM2 models. If the lengths of the signal, upstream, and downstream segments are *s*, *u*, and *d *respectively, then the corresponding conditional probabilities are given by:







If the length of a sequence is *L *= *u *+ *s *+ *d*, then the proposed MM1-SVM method is required to estimate *L*4^*k*+1 ^Markovian parameters, where *k *= 1. On the other hand, Loi-Rajapakse [32] is required to estimate,  Markovian parameters, where *k*_1_, *k*_2 _are the order of the Markov models having *k*_1 _= 2, and *k*_2 _= 1. It is shown that the output of a Multilayer perception (such as BPNN) is a polynomial of higher degree over the input variables [32]. It is also shown that the likelihood of a sequence given a model *M *can be approximated by a polynomial of conditional probabilities [32,39,40]:



### Classification of MM1 output

We applied SVM with polynomial kernel to classify MM1 encoded splice site data. Based on the training, a SVM can classify whether a query sequence contains an acceptor site or donor site. The splice site detection problem can be simplified into two binary-classification problems, one for acceptor sites and one for donor sites.

#### Support vector machines

The SVM is a canonical machine learning algorithm initially proposed by Vapnik [41-44]. It uses a hypothetical space of linear functions in a high dimensional feature space trained with a learning algorithm based on optimization theory. SVM classification is an optimization problem given by:





0 ≤ *α *_*i *_≤ *C*, *i = 1,...,l*,     (9)

where, *l *is the number of training examples, *k *is the kernel function, ***x ***is the input vectors, *y *is either -1 or +1 representing two different classes, ***α ***is the variable to be optimized and *C *is a trade-off parameter for generalization performance [41,42]. Each ***α ***corresponds to one particular training example and after the training process, only a subgroup of ***α ***will have non-zero values. This subgroup of ***α ***and their corresponding training examples are called the support vectors. In this study, two separate SVM classifiers are required, one for acceptor and one for donor. The class labels *y *in the two classifiers would then indicate true (*y *= +1) or false sites (*y *= -1) for acceptor and donor accordingly. Input ***x ***would always be a vector of MM1 probabilities.

Given a query DNA segment ***z***, the trained SVM classifies based on the decision function:



where *v *is the set of support vectors.

The kernel function in our classifiers is a second order polynomial:

*K*(***x***, ***z***) = (〈***x***·***z***〉 + 1)^2^,     (11)

where 〈·〉 indicates a dot product.

Expanding (11), we obtain



where *n *is the number of dimensions in vectors ***x ***and ***z***, and *x*_*i *_and *z*_*i *_are the i-th element in vectors ***x ***and ***z ***respectively. Substituting (12) into (10), the output *o*(*z*) becomes a second-order polynomial over ***z***, with the polynomial constants determined by *α *and ***x ***of the set of support vectors. Since ***z ***is a vector of conditional probabilities of a sequence of length *l*:

***z ***= [*P*(*S*_2 _| *S*_1_), *P*(*S*_3 _| *S*_2_),..., *P*(S_*l *_| *S*_*l*-1_)], (13)

the output *o*(*z*) in its polynomial form resembles equation (6). Such a polynomial of first order conditional probabilities suggests that a SVM classifier with the kernel function in (11) can approximate a higher order Markov model. Higher order polynomial kernels may also be used considering the trade-off of more complex decision function and larger training time. However, numerical instability often arises when higher order polynomial kernels are used.

#### Dataset

We have conducted several simulations to evaluate the performance of the proposed algorithm using two standard and publicly available splice site datasets.

**Figure 9:**
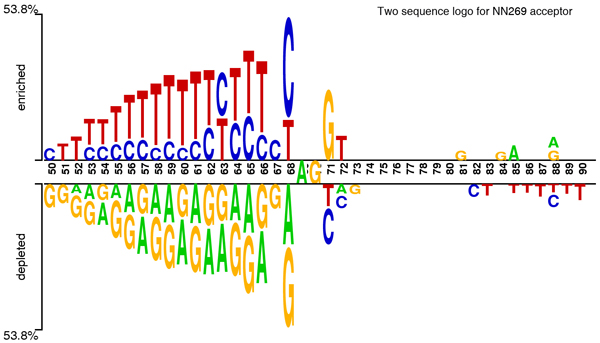
Two sample logo [46] of NN269 acceptor splice sites. It shows nucleotides which are enriched and depleted in the surrounding regions of the acceptor splice sites. The conserved dinucleotides AG is located in positions 69 and 70 in the sequence.

**Figure 10:**
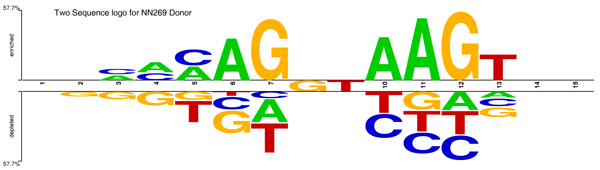
Two sample logo [46] of NN269 donor splice sites. It shows nucleotides which are enriched and depleted in the surrounding regions of the donor splice sites. The conserved dinucleotides GT is located in positions 8 and 9 in the sequence.

The first dataset is known as NN269 [18], which consists of 1324 confirmed true acceptor sites, 1324 confirmed true donor sites, 5552 false acceptor sites and 4922 false donor sites collected from 269 human genes. Each of the pseudo acceptor/donor sites also has AG/GT in the splicing junction but is not a real splice site according to the annotation. The window size for an acceptor is 90 nucleotides {-70 to +20} with consensus AG at positions -69 and -70. This includes the last 70 nucleotides of the intron and first 20 nucleotides of the succeeding exon. The donor splice sites have a window of 15 nucleotides {-7 to +8} with consensus GT at positions +1 and +2. This includes the last 9 bases of the exon and first 6 bases of the succeeding intron. The dataset is available at [45]. This data set is split into a training set and a testing set. The training data set contains 1116 true acceptor, 1116 true donor, 4672 false acceptor, and 4140 false donor sites. The test data set contains 208 true acceptor sites, 208 true donor sites, 881 false acceptor sites, and 782 false donor sites. Figure 9 and 10 show the two sample logo [46] of NN269 acceptor and donor sites. They represent the residues enriched and depleted in the sample. In NN269 acceptor dataset, AG is conserved in position 69 and 70 of the sequences, and for donor splice sites, GT is conserved in position 8 and 9 of the sequences.

We also used a second dataset named DGSplicer [3]. The DGSplicer true dataset is created by extracting a collection of 2381 real acceptor sites and 2381 real donor sites from 462 annotated multiple-exon human genes from [47]. Two of the donor splice sites and one acceptor splice site were excluded from the collection to form a set of 2380 real acceptor sites and 2379 real donor sites as those three splice sites contained symbols other than A, C, G, and T. Also a large collection of 400314 pseudo acceptor sites and 283062 pseudo donor sites were collected from 462 annotated human genes and used as the false dataset [3]. The window size for the acceptor is 36 nucleotides {-27 to +9} with consensus AG at positions -26 and -27, which includes the last 27 nucleotides of the intron and first 9 nucleotides of the succeeding exon. The donor splice sites have a window of 18 nucleotides {-9 to +9} with consensus GT at positions +1 and +2, which includes the last 9 bases of the exon and first 9 bases of the succeeding intron. The dataset is available at [48].

#### Model design

The splice site detection problem is divided into two sub problems, namely the acceptor splice site identification and the donor splice site identification. Two separate models are created for the identification of acceptor and donor splice sites. For example, for NN269 acceptor dataset, one MM1-SVM model is created and trained with NN269 acceptor training dataset (also refer to model learning section). To evaluate the classification performance of this model, the NN269 acceptor test dataset is used. Similarly a separate MM1-SVM model is trained and tested with NN269 donor training and donor test dataset.

#### Model Learning

The training of a model was conducted in two stages: the MM1 parameters estimation and the SVM with second order polynomial kernel training. The training sequences were aligned with respect to the consensus dinucleotides prior to stage one. The estimates of the MM1 are the ratios of the frequencies of each dinucleotide in each sequence position as shown in (14). Only the true splice site training sequences were used to create the Markov model. The desired output level is set to +1 or -1 depending on the true or false splice site class label.



We used the LIBSVM [49] implementation of the support vector machine, which is freely available at [50].

#### Model extension and comparison

To verify the usefulness of our proposed MM1-SVM method and to compare its performance with others, we also implemented several other methods that are closely related to the proposed method. We used different pre-processing scheme with a SVM and compare their performances. For instance, we combined a SVM with the zero order Markov model (MM0), which is also well known as WMM model. WMM assumes that the probability of observing a certain nucleotide at any position does not depend on the occurrence of any other nucleotides in any position of that sequence. A zero order WMM (i.e. WMM0) is obtained by counting the frequency of each nucleotide in each position. Similarly, higher order WMMs can be created by counting dinucleotides, trinucleotides etc. Literally MM0 and WMM0 are the same in terms of their working principle. In this study we have created several models including MM0-SVM, WMM0-SVM, MM1-SVM, and WMM1-SVM and we applied all the models in splice site identification.

#### Predictive accuracy measures

The classification performance is defined by the sensitivity (*S*_*N*_), specificity (*S*_*P*_), false positive ratio (FPR), and false negative ratio (FNR) of the model. The sensitivity, also known as true positive rate (TPR), is the percentage of correct prediction of true sites and specificity is the percentage of correct prediction of false sites. Specificity is the correct prediction of the false sites as defined below:



where, TP, TN, FP, and FN denote the number of true positives, true negatives, false positives, and false negatives (see Table 1) [29]. All the results in this paper refer to the canonical (GT/AG) splice sites leaving detection of the much less frequent (0.5–1%) non-canonical splice sites as a feature to be implemented in the future.

#### ROC analysis

Receiver operator curve (ROC) analysis is an effective and widely used method of assessing the performance of models [29]. It is a graphical representation of sensitivity and specificity of a classification model. ROC may also be created from the FPR and FNR of models [3]. When a ROC is created from the sensitivity (the y axis) and specificity (the x axis) of a model, the closer a curve follows the left-hand border and then the top of the border of the ROC plot, the more accurate the model [29] (refer to Figure 2, 3, 4 and 5). When the ROC is created from the FPR (on the y axis) and FNR (on the x axis) of the model, the closer a curve approaches the (0,0) point, the more accurate the model (refer to Figure 6 and 7).

#### Leave one out cross validation

A five fold cross validation technique is applied to determine the MM1-SVM splice site prediction accuracy and to compare the predictive accuracy with other standard published methods. The cross validation is performed by randomly partitioning the data into five independent subsets. Each of the subsets does not share any repeating sequences. Each model was trained by selecting four of the subsets (training data) and was tested on the fifth unused subset. Finally, we took the average of the five prediction accuracies as the final prediction measure of the model.

#### Proper window selection

Chen *et al*., [3] have conducted an extensive study for selecting a proper window size for the acceptor and donor splice site sequence. Based on the compositional characteristics of nucleotides and the presence of consensus in the sequence, they suggested an optimal length for the donor and acceptor splice site for the DGSplicer dataset. The study suggests a window from 9 bases upstream to 9 bases downstream (i.e.18 nucleotide) for exon/intron boundary best represents the donor splice site, and a window from 27 bases upstream to 9 bases downstream of the intron/exon (acceptor) best represents the acceptor splice site (i.e.36 nucleotides). For the DGSplicer dataset, we used the same acceptor and donor window length as suggested by [3].

## Conclusion

In this paper we presented a new method for the identification of eukaryotic gene splice sites. Unlike many existing methods, our proposed method is simple and effective. This method can be applied to identify splice sites in a large scale in newly sequenced genomes. Moreover this scheme can also be employed in the identification of other regulatory motifs in DNA sequences.

## Availability

Codes used in implementing the present method is freely available for academic use at [51]

## Authors' contributions

A. B. provided the conception and design of this study, implementation of the method as well as its analysis. B. C., S. K. H., and J. L. contributed to the design of the study and the interpretation of the results. All the authors contributed to the writing and critically revising the manuscript.

## Supplementary Material

Additional File 1 Additional file contains two figures showing the performance comparison of SVM with polynomial, linear and RBF kernels in terms of NN269 acceptor and donor dataset. It also contains two tables showing the sensitivity and specificity values of different methods. Click here for file

## Figures and Tables

**Table 1:** Definition of TP, TN, FP and FN

	Predicted positive	predicted negative
real positive	true positives, TP	false negatives, FN
real negative	true negatives, TN	false positives, FP
