# Correction: Splice site identification using probabilistic parameters and SVM classification

**DOI:** 10.1186/1471-2105-8-241

**Published:** 2007-07-05

**Authors:** AKMA Baten, BCH Chang, SK Halgamuge, Jason Li

**Affiliations:** 1 Dynamic Systems and Control Research Group, DoMME, The University of Melbourne, Victoria 3010, Australia

We proposed a method for the identification of splice sites [1] and it was tested against two data sets – DGSplicer (402695 acceptor and 285451 donor sites) and NN269 (6876 acceptor and 6316 donor sites).

The better performance on the bigger data set (DGSplicer) supports our claim about the validity of the proposed method. However, after the publication of this work it was brought to our attention that the results associated with the smaller dataset (NN269) in our paper were incorrectly scaled in the x-axis. The correct result generated by our proposed method (MM1-SVM) for this dataset is given in Figure 1 and Figure 2 below. This affects the results of Figures 2, 3, 4 and 5 of our original paper and Figures 1 and 2 in the additional file of the paper.

**Figure 1:**
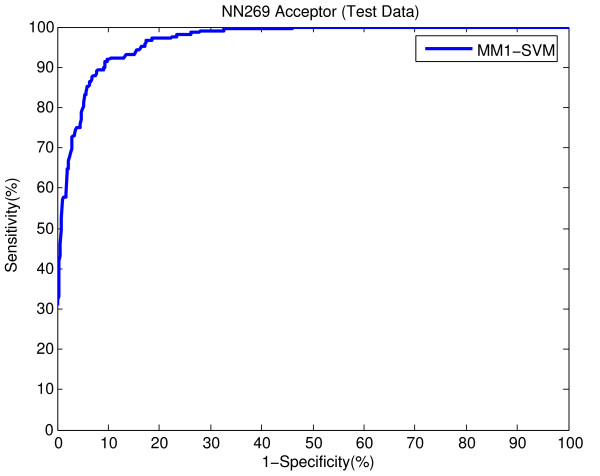
ROC showing the performance of MM1-SVM on NN269 acceptor splice site test data.

**Figure 2:**
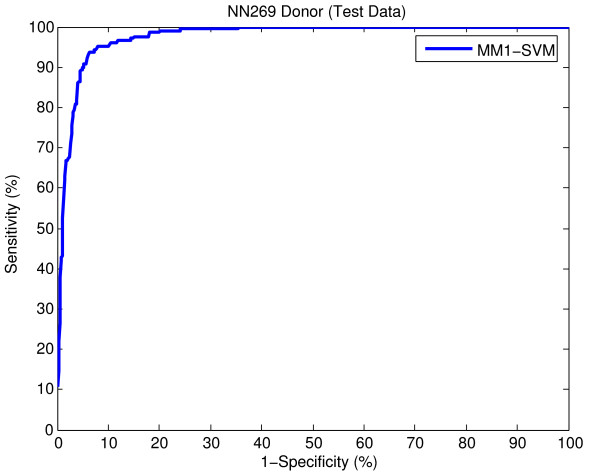
ROC showing the performance of MM1-SVM on NN269 donor splice site test data.

We regret any inconvenience caused by this incident and we would like to thank Dr. Gunnar Rätsch and Dr. Soeren Sonnenburg from Max Planck Society for bringing this error to our attention.
