# Supplementary material for: Splice site identification using probabilistic parameters and SVM classification
Source: BMC Bioinformatics. 2006 Dec 18;7(Suppl 5):S15. doi: 10.1186/1471-2105-7-S5-S15 (PMC1764471; doi:10.1186/1471-2105-7-S5-S15)
Supplement: Additional File 1 — Additional file contains two figures showing the performance comparison of SVM with polynomial, linear and RBF kernels in terms of NN269 acceptor and donor dataset. It also contains two tables showing the sensitivity and specificity values of different methods. [file 1471-2105-7-S5-S15-S1.pdf]

Figure 1: Comparison of performance between different SVM kernels for the identification of NN269 acceptor splice site. MM1 remains as the core pre-processing method while SVM with polynomial, linear and RBF kernel are used. All the kernels perform almost equally well.

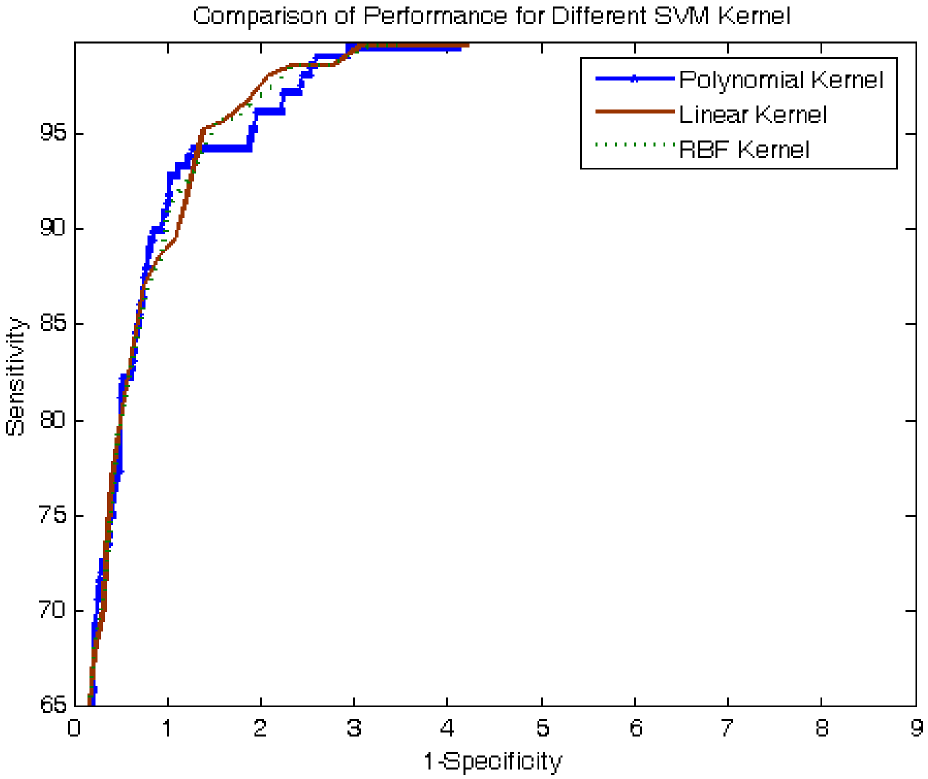

Figure 2: Comparison of performance between different SVM kernels for the identification of NN269 donor splice site. MM1 remains as the core pre-processing method while SVM with polynomial, linear and RBF kernel are used. All the kernels perform almost equally well.

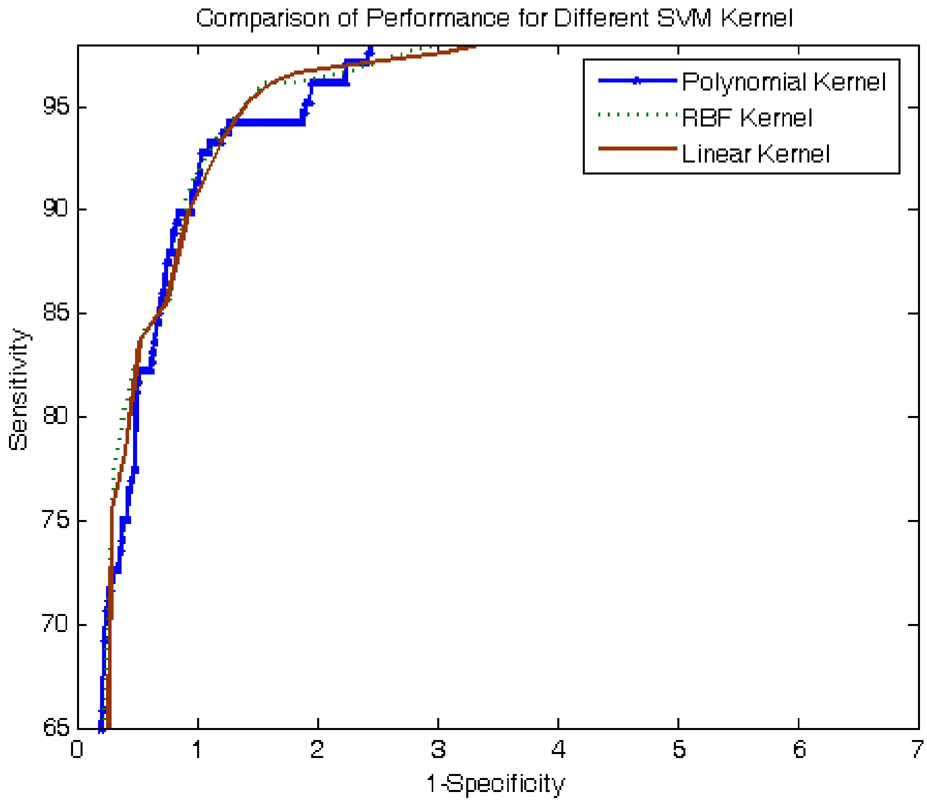

Table 1: NN269 Acceptor: the sensitivity and specificity values of MM1-SVM method and other methods. Sensitivity (TP/TP+FN), is the percentage of correct prediction of true sites and Specificity (TN/TN+FP) is the percentage of correct prediction of false sites.

SN	MM1-SVM 1-SP	Loi-Rajapakse 1-SP	GeneSplicer 1-SP	NNSplice 1-SP
65	0.21	0.9	0.7	1.1
70	0.28	1.1	0.9	1.3
75	0.41	1.2	1.7	1.6
80	0.55	1.5	2.6	2.3
85	0.75	2.8	3.4	3.6
90	1.1	4	5.5	4.8
95	2.0	5.9	7.9	10.4

Table 2: NN269 Donor: the sensitivity and specificity values of MM1-SVM method and other methods. Sensitivity (TP/TP+FN), is the percentage of correct prediction of true sites and Specificity (TN/TN+FP) is the percentage of correct prediction of false sites.

SN	MM1-SVM 1-SP	Loi-Rajapakse 1-SP	GeneSplicer 1-SP	NNSplice 1-SP
65	0.20	0.7	0.9	1.4
70	0.30	0.9	1.1	1.7
75	0.33	1	1.4	2.1
80	0.35	1.4	2.2	2.7
85	0.40	2.3	2.7	3.2
90	0.60	3	3.3	4.6
95	0.90	4.3	4.8	7.1

Table 3: Training time in seconds required for the SVM for NN269 and DGSplicer acceptor and donor splice site dataset.

**Dataset**	**Splice site**	**Training time (s)**
NN269	Acceptor	327
	Donor	109
DGSplicer	Acceptor	28891
	Donor	11921
